# Hemophagocytic Lymphohistiocytosis in a Patient With Human Immunodeficiency Virus

**DOI:** 10.7759/cureus.35139

**Published:** 2023-02-18

**Authors:** Talal Bazzi, Mark Benjamin, Nanaki Atal, Rabia Mahmood

**Affiliations:** 1 Internal Medicine, Ascension St John Hospital, Detroit, USA

**Keywords:** bone marrow failure, chemotherapy agents, hematological malignancies, hiv aids, hemophagocytic lymphohistiocytosis (hlh)

## Abstract

Hemophagocytic lymphohistiocytosis (HLH) is a rare and aggressive disorder that is often underdiagnosed due to its similarities in other forms of shock, most notably septic shock. In this case report, we discuss a patient who has a history of HIV presenting with altered mental status and cytopenias, ultimately going into shock and passing away. We initially thought we would be dealing with a case of septic shock, but a diagnostic workup during his hospital case lead to a diagnosis of hemophagocytic lymphohistiocytosis. This case illustrates how patients with HLH present very similar to septic shock and how to manage patients with this very aggressive disease.

## Introduction

Hemophagocytic lymphohistiocytosis (HLH) is a disorder of apoptosis resulting in an alteration of the regulatory pathways that terminate a body’s immune response which includes activation-induced cell death, anergy, and the induction of regulatory T cells. Consequently, a state of unchecked inflammation develops presenting with fevers, cytopenias, splenomegaly, and/or hepatomegaly, similar to septic shock [1]. The etiology of HLH may be categorized into primary (i.e., genetic causes) or secondary causes. In the adult population, secondary causes are more common and include infection, autoimmunity, and malignancy, specifically lymphoma [2]. Unfortunately, misdiagnosis is not uncommon given the similarities in clinical presentation to septic shock, and thus disease incidence is likely underrepresented. HLH is an aggressive disease and treatment modalities include immunosuppressive agents along with chemotherapy, with allogeneic hematopoietic stem cell transplant recommended in stable patients. In this case presentation we discuss a 46-year-old patient with HIV and a workup leading to the diagnosis of HLH.

## Case presentation

A 46-year-old African American male with a past medical history of HIV and on antiretroviral therapy presented to the hospital for altered mentation. Physical exam was notable for a disoriented patient with sinus tachycardia at a rate of 136 beats/min and tachypnea at 34 breaths/min. Chest X-ray and CT head were unremarkable and abdominal ultrasound showed hepatomegaly with no signs of the gallbladder or hepatic obstruction. Table 1 shows his labs on admission. Although afebrile on presentation, over the next 24 hours the patient spiked a fever with a Tmax of 103 °F and kidney function declined along with worsening transaminitis, and thrombocytopenia. Despite the initiation of broad-spectrum antibiotics, the patient became increasingly somnolent and he was transferred to the medical ICU given concerns over airway protection. The patient also developed coagulopathy with an international normalized ration (INR) of 1.91 and a partial thromboplastin time (PTT) of 61.0 seconds, and kidney function continued to decline with peak creatinine levels reaching 6.45 mg/dL. Hematology was consulted for further evaluation of thrombocytopenia, and the patient was eventually started on continuous renal replacement therapy (CRRT) due to refractory hyperkalemia in the setting of renal insufficiency and oliguria.

**Table 1:** Patient's lab values on admission and on day 3 of the hospital course ALT: alanine aminotransferase; AST: aspartate aminotransferase

	On admission	Day 3
Hemoglobin gm/dL	10.9	7.9
Platelet count K/mCl	91	58
Creatinine mg/dL	2.65	6.45
AST units/L	372	625
ALT units/L	237	225
Ferritin Value ng/mL	48,754	53,192
Triglyceride level mg/dL	299	
Soluble interleukin 2 (IL-2) pg/mL		58,776.3
Interleukin 6 (IL-6) pg/mL		480.8

**Figure 1:**
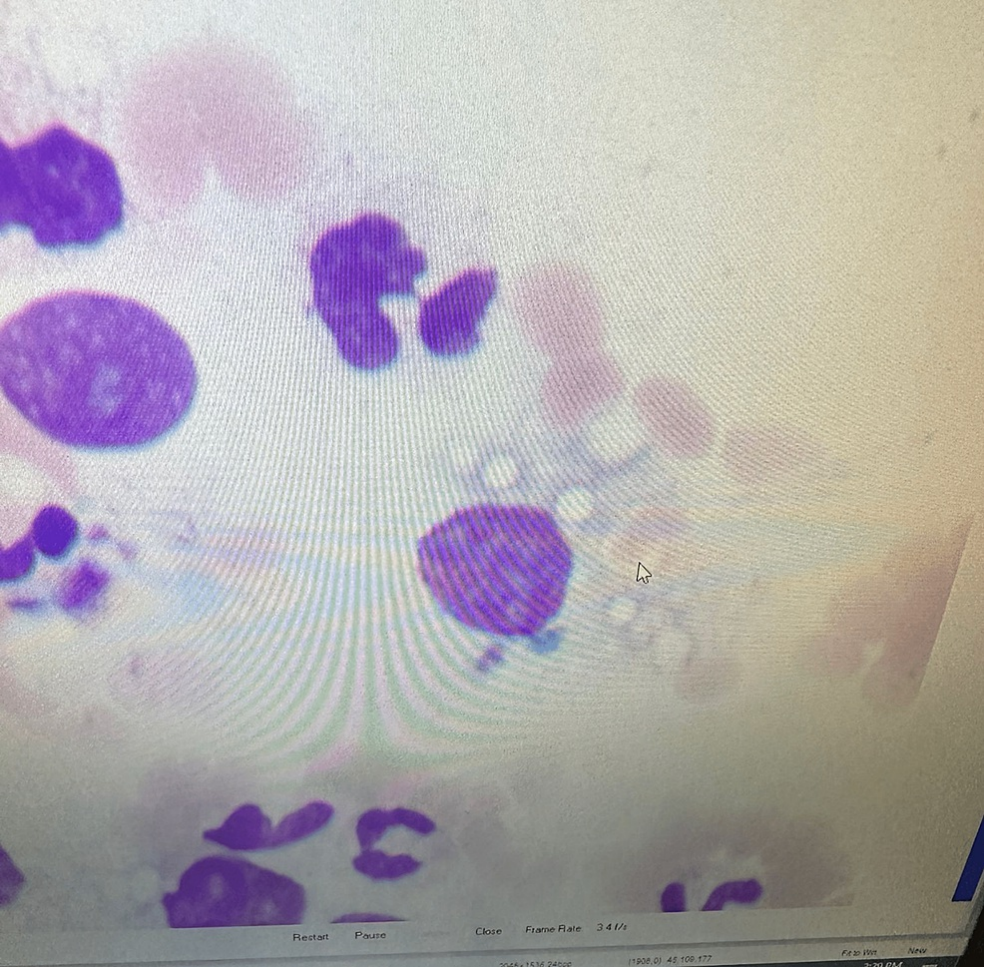
Diffuse histiocytic infiltration and histiocyte hyperplasia

On review, the patient's rapid clinical deterioration, cytopenias, liver function abnormalities, and neurologic symptoms raised suspicion for HLH, especially given the extreme elevation in the ferritin level. A bone marrow biopsy was emergently performed and yielded dysplastic erythropoiesis, dysplastic megakaryocytogenesis and progressive granulocytogenesis. Numerous histiocytes and focal hemophagocytosis were also noted as seen in Figure 1. Together, these features indicated HIV/AIDS myelopathy with hemophagocytosis. Soluble interleukin 2 (IL-2) and interleukin 6 (IL-6) levels were extremely elevated at 58776.3 pg/mL and 480.8 pg/mL, respectively. ​​​​​​

Over the next few days, the patient was intubated and started on immunosuppressive therapy with dexamethasone and chemotherapy with etoposide. Despite these interventions, pressors were needed for hemodynamic support, and the patient was ultimately transitioned to comfort care by his family. He passed away shortly thereafter.

## Discussion

HLH belongs to the family of diseases known as histiocytosis syndromes. It is extremely rare, with an incidence rate of 1.2 per 1 million people in patients under 18 years of age, and without known incidence in adults [3]. These patients are generally critically ill on presentation requiring pressor support and intensive care monitoring. Workup findings are notable for high fevers greater than 38.5℃, pancytopenia with remarkable thrombocytopenia, and multi-system organ failure. Unsurprisingly, clinicians usually follow a sepsis pathway initiating broad-spectrum antibiotics as HLH is not considered and goes undetected. 

As HLH progresses, the liver and spleen tend to be the most affected organs. Elevations in AST, ALT, and direct and indirect bilirubin levels develop as the disease process trends toward fulminant liver failure [4]. Acute renal insufficiency is also seen, with progressively increasing levels of creatinine and minimal urine output ultimately requiring emergent hemodialysis. Patients often succumb to the disease process due to refractory hypotension despite vasopressor support. The disease is so deadly because the ultimate treatment for this disorder is bone marrow transplantation, but patients are often too sick to undergo transplantation and therefore end up passing away from the disease process. 

The pathogenesis of HLH involves a sustained inflammatory reaction recognized as a cytokine storm. While the nidus for this process in primary HLH is not fully known, inciting factors in secondary HLH include infection, autoimmune process, and cancer, particularly hematologic malignancies (1,2). In a typical physiologic state, once these inciting factors are resolved, the immune system should revert to normal function with termination of cytokine production. In HLH, however, this fails to occur resulting in a mass cytokine production including interferon gamma, tumor necrosis factor alpha, IL-6, and IL-10 amongst others [5]. These cytokines are responsible for the pancytopenia, high fever, and multiorgan failure seen in patients with HLH [6].

Given the difficulties in identifying HLH, a standard diagnostic criterion was proposed in 2004 by Henter et al. [5]. The diagnostic criteria include a genetic mutation consistent with HLH or five of the following eight criteria: fever greater than 38.5℃ for more than seven days, splenomegaly, cytopenias affecting at least two cell lineages, hypertriglyceridemia above 265 mg/dL or a fibrinogen level under 1.5 g/L, ferritin more than 500 µg/L, hemophagocytosis in the bone marrow, soluble CD25 (sIL-2 receptor) more than 2400 u/mL, and low or absent natural killer (NK) cell activity [6]. Of note, these results are not inherently specific for HLH and may frequently be seen in septic shock. One key lab marker clinicians should look for in patients they are suspecting HLH is a ferritin level. A extremely high ferritin value can help aid in the diagnosis of HLH. Nevertheless, clinicians should be mindful of this criterion in cases refractory to broad-spectrum antibiotics and vasopressor support.

Treatment of HLH is often two-pronged, including immunosuppressive agents and chemotherapy. Immunosuppressive agents are usually given first to suppress the overactive immune system, thus diminishing the cytokine storm. This should help control the disease process, however, if it is unsuccessful, then cytotoxic anticancer drugs are needed. The most widely used therapy for HLH contains the chemotherapy drug etoposide and is known as the HLH-2004 therapy [6]. This therapeutic regime consists of ​​dexamethasone 10 mg/m² IV daily and etoposide every other day until the disease is controlled. If patients are being treated at centers with no chemotherapy, patients should at least be started on 10 mg/m² of dexamethasone. In addition to immuno-chemotherapy, if the patient is clinically stable, an allogeneic hematopoietic stem cell transplant is typically necessary. 

Despite intervention, the prognosis for patients with HLH is poor. After allogeneic stem cell transplants, the long-term survival is about 22% (7). One aspect affecting the prognosis includes the initiating disease factor. If the underlying cause is thought to be malignancy, the prognosis is more guarded as compared to an infectious or autoimmune cause. Higher levels of soluble IL-2 receptors are also considered a negative prognostic factor. 

Ultimately, HLH is an aggressive disease process that can be extremely difficult to diagnose and is associated with a poor prognosis. Early recognition is essential for prompt treatment with immunosuppressive and chemotherapeutic agents. HLH should be suspected if a patient has unexplained high fevers, cytopenias, and organ failure. A serum triglyceride, fibrinogen and ferritin level can help in the diagnosis, but more importantly, a bone marrow biopsy is needed to confirm the diagnosis. Finally, definitive treatment with an allogeneic hematopoietic stem cell transplant should be pursued if possible.

## Conclusions

HLH is a histiocytosis syndrome that frequently presents with critically ill patients requiring ICU-level care. Given the high mortality rate, early recognition, and initiation of treatment for the disease is crucial. Unfortunately, the constellation of symptoms including pancytopenia, fever, and multiorgan dysfunction are nonspecific and can masquerade as septic shock. This may delay definitive treatment, resulting in poor outcomes. Recognizing key details such as grossly abnormal liver and kidney function may guide a clinician to the correct diagnosis, but familiarity and utilization of the HLH-2004 criteria are imperative as that may expedite treatment. HLH mortality is extremely high, but with early recognition, familiarity with diagnostic criteria, and swift implementation of proper treatment, perhaps we can improve patient outcomes.
